# Screening, Genetic Variants, and Bipolar Disorders: Can Useful Hypotheses Arise from the Sum of Partial Failures?

**DOI:** 10.3390/clinpract13040077

**Published:** 2023-07-27

**Authors:** Mauro Giovanni Carta, Goce Kalcev, Alessandra Scano, Samantha Pinna, Cesar Ivan Aviles Gonzalez, Antonio Egidio Nardi, Germano Orrù, Diego Primavera

**Affiliations:** 1 Department of Medical Sciences and Public Health, University of Cagliari, Monserrato Blocco I (CA), 09042 Cagliari, Italy; maurogcarta@gmail.com (M.G.C.); samanthapinna1984@gmail.com (S.P.); diego.primavera@tiscali.it (D.P.); 2 Department of Surgical Sciences, University of Cagliari, Cittadella Universitaria, Blocco I, Asse Didattico Medicina P2, Monserrato (CA), 09042 Cagliari, Italy; alessandrascano@libero.it (A.S.); gerorru@gmail.com (G.O.); 3 Faculty of Health Sciences, Nursing Program, Univesidad Popular del Cesar, Sede Sabanas, Valledupar 20002, Colombia; infermiere2020@gmail.com; 4 Laboratory Panic and Respiration, Institute of Psychiatry (Ipub), Federal University of Rio De Janeiro (Ufrj), Rio De Janeiro 22725, Brazil; antonioenardi@gmail.com

**Keywords:** bipolar disorder, screener, MDQ, genetic risk, RS1006737, CACNA1C

## Abstract

Bipolar disorder (BD) is a relevant public health issue, therefore accurate screening tools could be useful. The objective of this study is to verify the accuracy of the Mood Disorder Questionnaire (MDQ) and genetic risk as screeners, and their comparison in terms of reliability. Older adults (N = 61, ≥60 years) received a clinical psychiatric evaluation, the MDQ, and were evaluated according to the presence of the genetic variant RS1006737 of CACNA1C. MDQ+ versus the diagnosis of BD as a gold standard shows a sensitivity of 0.286 (Cl 95% 0.14–0.39); a specificity of 0.925 (Cl 95% 0.85–0.08); a predictive positive value (PPV) of 0.667 (Cl 95% 0.33–0.91); and a predictive negative value (PNV) of 0.702 (Cl 95% 0.65–0.75). The positivity for the variant RS1006737 of the CACNA1C against the diagnosis of BD as a gold standard shows a sensitivity of 0.750 (Cl 95% 0.55–0.90); a specificity of 0.375 (Cl 95% 0.28–0.45); a PPV of 0.375 (Cl 95% 0.28–0.45); and a PNV of 0.750 (Cl 95% 0.55–0.90). The reliability between the MDQ+ and positivity for the variant RS1006737 of the CACNA1C was very low (K = −0.048, Cl 95% −0.20–0.09). The study found that both the genetic and the paper and pencil test were quite accurate, but were not reliable in case finding. In fact, despite some validity, albeit specular (in the case of a positive genetic test, the probability of having the disorder is very high, whereas in the case of a negative score on the paper and pencil test, the probability of not having the disorder is very high), the unreliability of the two tests (i.e., they certainly do not measure the same underlying dimension) opens the door to the need for an interpretation and the possibility of a synergistic use for screening. From a heuristic perspective, which obviously requires all of the necessary verifications, this study seems to suggest the hypothesis that a condition of hyperactivation common to disorders and stress conditions, and identified by a positive score on the MDQ (which is common to BD, post-traumatic stress disorder (PTSD), and anxiety disorders and whose genetic basis has not yet been clarified) can trigger BD in people with a predisposition to hyperactivity (i.e., in people with the condition identified by the analyzed genetic variant).

## 1. Introduction

Bipolar disorder (BD) is known to be a relevant public health issue and is one of the main causes of disability worldwide [1]. BD is associated with a high rate of comorbidity with other chronic diseases, a high risk of unemployment, and suicide [2]. The disorder is often recognized, on average, years later after the actual onset, which causes a delay in treatment and a worsening of the course [3]; thus, accurate screening tools could be useful.

In the first decade of the 2000s, a series of “paper and pencil” screener tests were developed [4], including the Mood Disorder Questionnaire (MDQ), the most famous and the most universally used in epidemiological surveys [5,6,7,8]. Compared to the epidemiological studies employing structured clinical interviews, those that employed the MDQ have identified a prevalence of the “spectrum” of bipolar disorders of 2–4% wider [4]. The accuracy of the MDQ has been the subject of controversy in subsequent years due to the excess of “false positives” detected by this screener [9]. In fact, in a clinical setting [9,10], patients who screened positive in the MDQ had not been diagnosed with BD by clinicians employing a semi-structured interview; conversely, they were diagnosed with post-traumatic stress disorder, specific phobia, alcohol and drug use disorders, impulse control disorder, eating disorders, and attention deficit disorder. Furthermore, in another survey, individuals who screened positive in the MDQ were also found by clinicians to have borderline personality disorders [11]. At the conclusion of this series of studies, the authors ended up recommending that the MDQ is not useful for case finding in clinical practice in the two-phase epidemiology studies [12].

In contrast to this perspective, it was noted that people who scored “positive” on the MDQ but who were not affected by BD were frequently homogeneous with BD [4] in terms of: (a) demographic characteristics, such as gender and age [13]; (b) functioning and disability status such as high level of distress, low social functioning, low employment rate [6,13], and a lower quality of life compared to the individuals with the same diagnosis; e.g., simple phobia without a positive screen on the MDQ [14,15]; (c) clinical features: the clinical features of the disorders of the individuals who scored positive on the MDQ but who were not affected by BD are known to be frequently associated with BD, i.e., borderline personality disorder [16,17]; post-traumatic stress disorder [18,19]; phobic disorder [20,21]; alcohol use disorder [22,23]; substance use disorder [24]; eating disorder [25,26,27]; impulse control disorder [28]; and attention deficit disorder [29]; (d) the intensity of recourse to treatments: the individuals scoring positive on the MDQ but who were not affected by BD show heavy recourse to health care, use of mood stabilizers and antidepressants. While none of these components can be individually considered pathognomonic of BD, their co-occurrence could identify “sub-threshold conditions of BD” or early antecedents in individuals that may later develop BD. It is well known that the diagnosis of BD generally occurs even ten years after other clinical manifestations; anxiety, eating, and substance use disorders preceded bipolar disorders in around 50% of cases [30].

New evidence has recently been added to this controversy, favoring the poor validity of the MDQ since, according to the authors, the MDQ score does not correlate with a pattern that is closely linked to BD, such as genetic risk or the presence of genetic variants associated with BD. On the contrary, MDQ positivity was found to have a higher genetic correlation with post-traumatic stress disorder, attention deficit hyperactivity disorder, insomnia, and major depressive disorder [31]. The authors suggest that MDQ may capture symptoms of general distress or psychopathology rather than hypomania/mania, specifically in at-risk populations [31]. However, these results contrast with those from another study that found a strong association between the MDQ score and a (different) pool of genetic risk factors [32]. An explanation for these contrasting results may be that the pools of genetic variants analyzed in the studies may not be the expression of a single risk, and that the disorder (of which the common syndrome is known, but a common pathogenesis is not) may be the result of the interaction of complex (and even non-unique) risk pathways. In this framework, a single variant may be the expression of a specific vulnerability requiring interaction with other factors for the manifestation of the disorder as a result of complex interactions.

Our recent line of research has shown that a specific variant associated with BD (CACNA1C rs1006737) was detectable in hyperactive elderly people without BD to an equal extent than in elderly people with BD, while non-hyperactive elderly people without BD had a much lower frequency of the variant in question [33,34,35].

Thus, although the two “case finding tools” (the CACNA1C variant and the MDQ) are both related to BD, they were found to be inadequate to identify the disorder with accuracy: the CACNA1C variant is actually common not only to the disorder, but also in people with hyperactivity without the disorder [33,34,35]. Whereas, the MDQ, as it is not specific to BD, would identify a non-specific risk common to other disorders as well [9,10]. Thus, it could be useful to verify whether these two risk factors are superimposable or synergistic in the identification of bipolar disorders. In this case, it could be hypothesized that they are the expression of different potentially synergistic risk factors.

As a result of the need for a hypothesis, small sample analyses and the use of mixed methods can lead to interesting findings.

To add some new evidence to this perspective, we used the same sample of elderly people from our previous research to verify whether the MDQ can be an accurate screening tool for BD, and whether the presence of the CACNA1C gene and the genetic variant RS1006737 can be considered as an accurate screener for BD. We also compared the two screeners for BD in terms of reliability.

## 2. Materials and Methods

### 2.1. Design of the Study

A cross-sectional study, in which the accuracy of a positive score on the Italian version of the MDQ at a cut-off of ≥8 [7] was assessed against the diagnosis of BD, was carried out by expert psychiatrists that employed the Structured Clinical Interview for DSM-IV Axis I Disorders (SCID) [36] as a gold standard. A similar method was used to establish whether the presence of the CACNA1C gene and the genetic variant RS1006737 can be used as a screener for BD, as the latter has been frequently associated with a genetic risk for BD [37,38]. The reliability of a positive score on the MDQ and the presence of the variant RS1006737 of the CACNA1C gene as screeners for BD was then assessed.

### 2.2. Participants

The study sample included 61 older adults (60 years or older) who had been involved in a study that measured the frequency of the CACNA1C gene and of the genetic variant RS1006737 in people with hyperactivity and without BD. The exclusion criteria included severe health conditions. A total of 40 participants in the sample included individuals from a previous survey on active aging in the elderly who had never been diagnosed with lifetime bipolar spectrum disorder [39,40]. A control group of older adults with a diagnosis of BD who attended the Centro di Psichiatria di Consultazione e Psicosomatica of the University Hospital of Cagliari, Italy was also included in the study (21 participants). The exclusion criteria of the above cited previous study excluded cognitive deficits, substance abuse disorders and severe chronic disorders. Mean inflammatory indices were normal at the end of the randomized control trial (RCT) [41]. Of the total sample of people without BD, 10% had type 2 diabetes; 40% had mild-medium hypertension; 10% had previously had a diagnosis of neoplastic disease, which was in remission [42]; and 10% had at least grade I obesity [43]. Out of the 21 patients with BD, 7 (33%) suffered from at least I degree obesity (body mass index (BMI) ≥ 30), 3 (14.%) from type II diabetes, and 6 (29%) from moderate or mild hypertension.

### 2.3. Psychiatric Evaluation

Both groups underwent psychiatric evaluation, including psychiatric interviews and the administration of the Mood Disorder Questionnaire (MDQ).

### 2.4. Genotyping

Genotyping was conducted at the Molecular Biology Laboratory of the Department of Surgical Sciences at the University of Cagliari, Italy. It consisted of a preanalytical phase in which the blood sampling of all 61 participants underwent fluorescent probes, Frequency Resonance Energy Transfer (FRET) and oligonucleotides for polymerase chain reaction (PCR-PRIMER) to detect polymorphisms [44]. In the analytical phase, the genomic deoxyribonucleic acid (DNA) was extracted by means of the Bosphore Viral DNA/RNA Extraction Spin Kit (Anatolia gene works, Turkey). The concentration and the purity of the extract were verified by means of the NanoDrop spectrophotometric system (Thermo Fisher Scientific, Monza, Italy). The real-time PCR instrument amplified the examined DNA fragment. Upon PCR completion, the probe DNA system was heated at constant velocity. For quality control purposes, all samples were also analyzed using the Sanger sequencing method [44]. In the post-analytical phase, data were processed and compared through Blast software (https://blast.ncbi.nlm.nih.gov/Blast.cgi/ (accessed on 21 November 2022)), and Clustal W (https://www.ebi.ac.uk/Tools/msa/clustalo (accessed on 21 November 2022)) [44].

### 2.5. Ethical Considerations

This study’s protocol has been approved by the Comitato Etico Indipendente of the Azienda Mista Ospedaliero Universitaria di Cagliari (NP 2893/2022 of 11 July 2022). All of the subjects participating in this research have provided written informed consent.

### 2.6. Data Analysis

The accuracy of MDQ positivity and positivity for the variant RS1006737 of the CACNA1C gene was measured in terms of Sensitivity, Specificity, Predictive Positive Value (PPV), and Predictive Negative Value (PNV), using the clinical diagnosis of BD as a gold standard. The reliability of MDQ positivity and positivity for the variant RS1006737 of the CACNA1C gene was measured by means of the K value.

## 3. Results

Table 1 shows the characteristics of the whole sample of people aged 60 years and older. The distribution by sex was homogeneous.

Table 2 shows the accuracy of MDQ+ versus the diagnosis of BD: the sensitivity was 0.286 (Cl 95% 0.14–0.39); the specificity was 0.925 (Cl 95% 0.85–0.08); the PPV was 0.667 (Cl 95% (0.33–0.91); and the PNV 0.702 (Cl 95% (0.65–0.75).

Table 3 shows the accuracy of the positivity for the variant RS1006737 of the CACNA1C gene as a screener versus the diagnosis of BD: the sensitivity was 0.750 (Cl 95% 0.55–0.90); the specificity was 0.375 (Cl 95% (0.28–0.45); the PPV was 0.375 (Cl 95% 0.28–0.45); and the PNV was 0.750 (Cl 95% 0.55–0.90).

Table 4 shows the reliability between the positivity for the variant RS1006737 of the CACNA1C gene and the positive score on the MDQ; the K Cohen value was found = −0.048 (Cl 95% −0.20–0.09).

## 4. Discussion

Both of the examined “screening tools” appear to be insufficient. However, it is surprising that they present complementary performances; although the MDQ positivity shows excellent specificity (0.925), the variant positivity shows sufficient sensitivity (0.750). It is therefore interesting that their reliability is very low. This is consistent with the hypothesis that each of them may be the expression of a different component of the disorder.

The low sensitivity of the MDQ is apparently surprising: although the screener has often been criticized for its high number of false positives, in this sample it shows a high number of false negatives (15/21 = 71.4%). However, this discrepancy from the data reported so far can be easily explained by taking into consideration the specificity of the sample examined, since it included elderly people who would be highly unlikely to develop BD in the future. It is therefore extremely unlikely in such a sample to find people with anxiety symptoms or borderline personality traits who “in the future” will be affected by BD. The low frequency of false positives in this sample of elderly people could therefore suggest that the hypothesis that the false positives identified by the MDQ are people with a subthreshold condition at risk of manifesting a frank bipolar pathology “in the future” is true.

The specificity of the MDQ in the third sample may also explain the high number of false negatives. In fact, it is known that many bipolar disorders in old age result in chronic depression [45]. This is in line with the neo-Kraepelinian view of the priority of mania in mood disorders, that views depression as the ashes after the fire of mania [46]. It is easy to understand how clinical manifestations that become longstanding chronic depressions can more frequently give rise to recall bias in relation to the times of early hyperactivity. These considerations do not apply to all elderly people diagnosed with BD, but they certainly do to a large proportion. Furthermore, this consideration would explain the decrease in the MDQ prevalence found in the elderly segments of the general population in community surveys in several countries [13,47]. This phenomenon is an apparent paradox if we consider the addition of lifetime diagnoses over periods of age. These considerations suggest that the MDQ cannot be used in elderly people, and only a strong recall bias can explain it. The same recall bias in the lifetime diagnosis of periods of hypomania in elderly people diagnosed with BD would explain the high frequency of false negatives that we have found on the MDQ.

In a diametrically opposite way, the genetic screener has insufficient specificity. As the previous works on the same sample demonstrated, several individuals who were not affected by BD but who had traits of hyperactivity and novelty-seeking without bipolar pathologies and were well-integrated into the social context [33], were positive for the variant under examination [35]. On the other hand, the high sensitivity indicates that among those who were affected by BD, a large percentage was positive for the genetic variant under examination.

However, we must still emphasize that the intent was not to test a single gene adopting it as a potential screener. Rather, since it had been verified that the gene is associated with BD, but that it was also found in healthy people without BD but with adaptive hyperactivity (therefore positive tests could result in BD or being healthy), we aimed to verify if this could be synergistic with what was identified by the MDQ test (where both people with BD and people with other disorders obtained positive scores).

Gene variant identification has yielded promising results in BD biology, particularly in the identification of genes coding for calcium channel subunits such as CACNA1C [48]. The gene CACNA1C (Calcium Voltage-Gated Channel Subunit Alpha1 C) is located in locus 12p13.33. It encodes an Alpha-1 subunit of a voltage-dependent calcium channel, and the suspect single nucleotide polymorphism (SNP) associated with BD is the rs1006737 allele A [49]. An association was found between calcium channel genetics (linked with variants of CACNA1C) and increased glutamatergic metabolites in BD, possibly playing a synergic role in intracellular Ca^2+^ overload and excitotoxicity [50]. In fact, there are different considerations about the role of the mutation rs1006737 in mood disorders. Some authors showed no or poor association [49], while others remarked on an interesting association between this SNP and mood or cognitive dysfunctions [51]. In following the “clinical road” exclusively, the result is strongly influenced by different factors such as the patient cohort used, i.e., number of patients, age, and in particular, the methodology to diagnosis, executed in different clinical reports with different approaches [51]. Furthermore, these studies did not report the role of other genome mutations able to interfere with the primary function of this SNP [52]. However, an exhaustive representation of the mutation rs1006737 should be integrated with reconstruction experiments in animal models, or in vitro by cell cultures able to define the impact of mutation function without other biological interferences following an approach of “translational genomics” for mood disorders [53]. This mutation has been associated with an odds ratio of 1,7 for BD, according to the clinical genetics database SNPedia. At the same time, this database associates this mutation with a trait for autism spectrum disorder, attention-deficit/hyperactivity disorder, bipolar disorder, major depressive disorder, and schizophrenia—in particular, reporting different publications through genome-wide association studies (GWAS) [54].

However, the same variants have also been found to be associated with schizophrenia and not just BD [55]. Current methods estimate that the cumulative impact of many frequent alleles may explain only 38% of the BD phenotypic variance [56]. The considerations made so far lead us to affirm that it is not only incorrect and dangerous to think that the presence of a genetic well-known determinant can identify the presence of BD, but also that it is strongly distorting to think of adopting the genetic well-known determinants as a “gold standard” in identifying bipolar disorders. This operation seems to repeat some traditional mistakes of psychiatry that erroneously translated simple, unproven hypotheses into scientific truths, including the terms psychosis and neurosis that arose on the basis of the (unproven) hypothesis that mental disorders were of a degenerative nature. Likewise, the concept of a schizophrenogenic mother was developed on the basis of the (unproven) hypothesis that it was an incorrect way of relating to her son that produced schizophrenia. Similar biases could arise if one misidentifies the presence of a genetic trait that has been found to be associated with the disorder itself.

In contrast, the questionnaires (especially the MD) were proved to be inaccurate, particularly in relation to the clinical diagnosis assumed to be the gold standard. This is probably because clinicians analyze: (1) what patients say; (2) what they see themselves; and (3) what key informants (such as the wife or husband) say with the patient’s consent. This is then used as a source of information (this is important, especially in bipolar II disorder where there is often little awareness by the patient) [4]. Of these three sources, the questionnaires use only the first one. Nonetheless, the questionnaires have shown some accuracy, albeit this is not sufficient [4].

At the end of this analysis, the low accuracy found between the genetic test and the paper and pencil test demonstrate the inadequacy of both as screeners. In fact, despite some validity, albeit specular (in case of a positive genetic test the probability of having the disorder is very high, whereas in the case of a negative score on the paper and pencil test, the probability of not having the disorder is very high), the unreliability of the two tests (i.e., they certainly do not measure the same underlying dimension) opens the door to the need for an interpretation. Therefore, there is the possibility of a synergistic use for screening.

Psychiatrists hope that biological markers (e.g., the objective measurement of pathophysiological processes involved in disease development) will soon be used and will enable the prediction of disease course, prognosis, and treatment efficacy. However, considering the decades of “promising” genetic research, the results to date are disappointing. Hopes for the future do not allow us to address the current problem of great concern in terms of public health, since BD is often diagnosed years later. Perhaps it could be useful to generate pathogenetic hypotheses about how different factors can interact in complex pathogenic pathways. Since we start from a descriptive classification, it is possible that the same syndrome is the result of different biological mechanisms, or that some conditions can identify potential synergistic risk factors in producing the disorder in question. From this point of view, an analysis with mixed methods and on a small sample size can produce pathogenetic hypotheses, without which the search for biomarkers can be reduced to throwing fishing nets. In this framework, and only from a heuristic perspective (which obviously requires all the necessary verifications), this small study seems to suggest the hypothesis that a condition of hyperactivation common to many disorders and identified by the positivity to the MDQ (which is common to BD, PTSD and anxiety disorders [9,10,11,12] and whose genetic basis has not yet been clarified) can trigger BD in people with a predisposition to hyperactivity (i.e., in people with the condition identified by the analyzed genetic variant).

### Limitations

This study is on a small and specific sample whose data concern only one genetic variant. However, the study was developed solely to generate heuristic hypotheses according to a mixed methods analysis perspective. From this point of view, the specificity of the sample of older adults who are unlikely to obtain positive scores in the future if identified without BD is important.

## 5. Conclusions

The study found that both the genetic and the paper and pencil test were accurate, but they were not reliable if used as screeners. In fact, despite some validity, albeit specular (in case of a positive genetic test, the probability of having the disorder is very high, whereas in the case of a negative score on the paper and pencil test, the probability of not having the disorder is very high), the unreliability of the two tests (i.e., they certainly do not measure the same underlying dimension) opens the door to the need for an interpretation and the possibility of a synergistic use for screening. From a heuristic perspective, which obviously requires all of the necessary verifications, this study seems to suggest the hypothesis that a condition of hyperactivation common to disorders and stress conditions and identified by a positive score on the MDQ (which is common to BD, PTSD, and anxiety disorders and whose genetic basis has not yet been clarified) can trigger BD in people with a predisposition to hyperactivity (i.e., in people with the condition identified by the analyzed genetic variant).

This study also demonstrates how the choice of a screener should fit the framework, i.e., the performance of the MDQ in old age seems completely different from that recorded at other ages.

## Figures and Tables

**Table 1:** Characteristics of the sample.

		People with Bipolar Disorder	People without Bipolar Disorder
		N = 21	N = 40
**Gender**	Man	7 (33.4%)	22 (55%)
	Woman	14 (66.6%)	18 (45%)

Chi square = 2.58 p = 0.107, OR = 0.41 CI 95% (0.12–1.23).

**Table 2:** Accuracy of MDQ+ versus Diagnosis of Bipolar Disorder.

	Bip+	Bip−
MDQ+	6	3
MDQ−	15	37

Sensitivity 0.286—Cl 95% (0.14–0.39); Specificity 0.925—Cl 95% (0.85–0.08); PPV 0.667—Cl 95% (0.33–0.91); PNV 0.702—Cl 95% (0.65–0.75).

**Table 3:** Accuracy of Gen+ versus Diagnosis of Bipolar Disorder.

	Bip+	Bip−
GEN+	15	25
GEN−	6	15

Sensitivity 0.750—Cl 95% (0.55–0.90); Specificity 0.375—Cl 95% (0.28–0.45); PPV 0.375—Cl 95% (0.28–0.45); PNV 0.750—Cl 95% (0.55–0.90).

**Table 4:** Concordance of Gen+ and MDQ+.

	MDQ+	MDQ−
GEN+	5	35
GEN−	4	17

K = −0.048 Cl 95% (−0.20–0.09).

## Data Availability

The data presented in this study are available on reasonable request from the corresponding author. The data are not publicly available due to privacy restrictions.
